# Blood cell markers that can predict the long-term outcomes of patients with colorectal cancer

**DOI:** 10.1371/journal.pone.0220579

**Published:** 2019-08-01

**Authors:** Hironori Mizuno, Norihiro Yuasa, Eiji Takeuchi, Hideo Miyake, Hidemasa Nagai, Yuichiro Yoshioka, Kanji Miyata

**Affiliations:** Department of Gastrointestinal Surgery, Japanese Red Cross Nagoya First Hospital, Michishita-cho, Nakamura-ku, Nagoya, Japan; McGill University, CANADA

## Abstract

**Objectives:**

To identify blood cell markers that predict the long-term outcomes of patients with colorectal cancer.

**Methods:**

Data from 892 stage II and III colorectal cancer patients who underwent R0 resection were included. We analyzed the correlations of the preoperative blood data, previously reported prognostic indices, and clinicopathologic factors with the long-term outcomes, such as relapse-free survival and overall survival, using univariate and multivariate analyses.

**Results:**

Multivariate analysis showed that tumor location, stage, mean corpuscular volume, neutrophil-to-lymphocyte ratio, and lymphocyte-to- monocyte ratio were significantly correlated with relapse-free survival. A mean corpuscular volume ≥80.5 fL, neutrophil-to-lymphocyte ratio ≥5.5, and lymphocyte-to- monocyte ratio <3.4 had hazard ratios for disease relapse between 1.39 and 1.93. The cumulative scores of these three factors were aggregated into a laboratory prognostic score, with a maximum score at 6. The relapse-free survival and overall survival were well stratified by a laboratory prognostic score between 0–3 and 4–6, respectively, independent of the stage.

**Conclusion:**

The mean corpuscular volume, neutrophil-to-lymphocyte ratio, and lymphocyte-to- monocyte ratio can serve as blood cell markers to predict the long-term outcomes of patients who underwent R0 resection for stage II/III colorectal cancer.

## Introduction

Colorectal cancer (CRC) is the third most common cancer in men, the second most common cancer in women, and the fourth most common cause of cancer-related deaths worldwide [1]. Curative resection is the gold-standard treatment for non-metastatic CRC. However, despite the advances in the preceding decades, CRC survival remains unsatisfactory. Therefore, it is critically important to optimize the treatment, including the use of adjuvant therapies, for curatively resectable disease by identifying patients at greater risk for worse outcomes. Pathologic stage is the most important prognostic indicator for CRC; however, recently, there had been increasing interest in improving CRC prognostication using routine blood test data. Although there had been many studies that investigated several biomarkers, the results were inconsistent [2–15]. Notably, biomarkers that are reliable and easy to obtain are required to implement adequate relapse risk and survival assessments. Therefore, the aim of the present study is to identify blood cell markers that can prognosticate the long-term outcomes of CRC patients, independent of tumor stage.

## Materials and methods

### Patients

We reviewed a prospectively recorded database of stage II and III CRC patients who underwent elective R0 resection at the Japanese Red Cross Nagoya First Hospital, Nagoya, Japan, between January 2003 and December 2013. Patients who underwent chemotherapy or radiotherapy before surgery or those who underwent R1 or R2 resection were excluded. A total of 892 patients were identified and included in the study. The mean patient age was 68.6 ± 10.3 years (range, 19–93 years), and 57.3% were men. In accordance with the Japanese Society for Cancer of the Colon and Rectum guidelines [16], postoperative adjuvant chemotherapy (PAC) was administered for stage III patients and stage II patients at high risk for recurrence (i.e., T4, perforation, poorly differentiated adenocarcinoma, vascular invasion, and bowel obstruction), with informed consent. After exclusion of patients who refused, 39 (9%) stage II and 232 (52%) stage III patients received PAC. The most frequently used PAC regimens were uracil/ tegafur and oral leucovorin, uracil/ tegafur, XELOX, capecitabine, and S-1.

### Methods

The preoperative routine blood test data obtained within 2 weeks before surgery included white blood cell count, neutrophils (%), lymphocytes (%), monocytes (%), red blood cell (RBC) count, hemoglobin concentration (Hb), mean corpuscular volume (MCV), red cell distribution width (RDW), platelet count, neutrophil-to-lymphocyte ratio (NLR), lymphocyte-to- monocyte ratio (LMR), and platelet-to-lymphocyte ratio (PLR). In addition, C-reactive protein (CRP), serum albumin, total cholesterol (T-Chol), Glasgow prognostic score (GPS), controlling nutritional status (CONUT) score, and Onodera’s prognostic nutrition index (PNI) were investigated as the conventional prognostic scores that reflected systemic inflammatory, nutritional, and immunologic status [7, 14, 15]. The GPS comprised high CRP level (>1.0 mg/dL) and hypoalbuminemia (<3.5 g/dL); patients with both criteria were allocated a score of 2, those with only 1 criterion were allocated a score of 1, and those without abnormalities were allocated a score of 0 [7]. PNI was calculated as follows: 10 × albumin value (g/dL) + 0.005 × lymphocyte of the peripheral blood. A higher value suggested good immune-nutritional status [14]. CONUT score was calculated from three parameters, serum albumin, T-Chol, and total peripheral lymphocyte count; a lower value indicated favorable nutritional status [15]. The cutoff values of the continuous variables were determined by receiver operating characteristic (ROC) curve analysis for discriminating relapse/non-relapse in patients who were followed up for more than 3 years. In the ROC analysis, the optimal cutoff values were determined to be the point where the vertical distance between the ROC curve and the diagonal line was maximal.

The resected CRCs were histopathologically classified according to the seventh edition of the UICC/TNM classification [17]. Our surgical department followed a standardized surveillance protocol, wherein patients were followed up by clinical assessment every 3 months for 5 years. The collected postoperative data included clinical assessments, laboratory tests, and computed tomography of the chest, abdomen, and pelvis. All evidences of disease recurrence were obtained from the patients’ medical records. The disease relapse was determined by imaging modalities including CT and FDG-PET, physical findings and blood tests including serum CEA and CA19-9. Follow-up information through July 2017 was compiled for all survivors. The RFS was calculated based on the time from the date of surgery to the date of identification of disease relapse, whereas the overall survival (OS) was based on the duration from surgery to death due to any cause.

We attempted to identify the prognostic factors from the blood data and the previously reported prognostic scores that had a significant relationship with RFS and OS using univariate and multivariate analyses. The study protocol was approved by the Ethics Review Committee of Japanese Red Cross Nagoya First Hospital (approval number: 2018–080), which waived the need for informed consent due to the retrospective nature of the study.

### Statistical analysis

Continuous variables were expressed as mean ± SD or median, IQR, and were compared using student t-test. Differences in categorical variables were compared using chi-square test. The Kaplan–Meier method was used to estimate survival curves, whereas the log-rank test was employed to evaluate the differences in survival between groups. Considering the statistical significance and clinical implications of the univariate analysis, variables were entered into a multivariate analysis to identify the significant independent prognostic factors of RFS and OS. The neutrophil (%), lymphocyte (%), and NL R were integrated into NLR, because they had a close relationship, and the AUC of the NLR was larger than that of neutrophil (%) and lymphocyte (%). Similarly, RBC count and Hb were integrated into Hb. Hazard ratio (HR) and 95% confidence interval (CI) were calculated during multivariate analysis using a Cox proportional hazard model. To develop an easy scoring system for RFS, a laboratory prognostic score (LPS) was calculated based on the log of the regression coefficient of the factors [18]. According to the method described by Moons KG [18], the natural logarithm of the hazard ratio in Cox proportional hazard model (the regression coefficients) was multiplied by 10, and rounded to the nearest integer to obtain easily applicable scores per predictor. An MCV ≥ 80.5 fL, NLR ≥ 5.5, and LMR < 3.4 were scored 3, 2, and 1, respectively. Statistical analyses were performed using JMP version 10.0 for Windows (SAS Institute Inc., Cary, NC, USA) at a significance level of p < 0.05.

## Results

The patient demographics are presented in Table 1. Five patients died from postoperative complications (operative mortality rate = 0.56%). The median follow-up duration was 58.7 months (interquartile range, 28.2–86.9 months).

**Table 1:** Patients demographics.

Age		68.6±10.3		
Sex (M:F)		511:381		
Tumor location				
	Cecum	69		(8%)
	Appendix	7		(1%)
	Ascending colon	145		(16%)
	Transverse colon	96		(11%)
	Descending colon	58		(7%)
	Sigmoid colon	241		(27%)
	Rectum	271		(30%)
Histological grade				
	well differentiated	59		(7%)
	moderately differentiated	766		(86%)
	poorly differentiated	12		(1%)
	other	55		(6%)
T				
	1	13		(2%)
	2	47		(5%)
	3	690		(77%)
	4	142		(16%)
N				
	0	448		(50%)
	1	324		(36%)
	2	102		(11%)
Stage				
	II	448		(50%)
	III	444		(50%)
Preoperative laboratory data				
	C-reactive protein (mg/dL)	1.15±2.42		(0–24.7)
	Albumin (g/dL)	3.75±0.6		(1.2–4.9)
	Total cholesterol (mg/dL)	176.1±41.8		(65.0–410.0)
	White blood cell count (x10^9^/L)	6.45±2.21		(1.9–19.5)
	Neutrophil (%)	63.9 (56.8–71.2)		(20.1–96.9)
	Lymphocyte (%)	25.1±9.1		(1.9–69.4)
	Monocyte (%)	7.8±2.7		(1.1–29.3)
	Red blood cell count (x10^9^/μL)	3.98 (3.61–4.34)		(2.09–6.00)
	Hemoglobin (g/dL)	11.6±2.0		(5.8–17.5)
	Mean corpuscular volume (MCV)(fl)	88.2±8.5		(59.2–118.5)
	Red cell distribution width (RDW)	16.9±5.5		(0–39.6)
	Platelets count (x10^9^/L)	275.8±101.8		(39.0–1221.0)
	Neutrophil-to-lymphocyte ratio (NLR)	3.25±2.80		(0.34–51.0)
	Lymphocyte-to-monocyte ratio (LMR)	3.58±2.58		(0.54–63.09)
	Platelets-to-lymphocyte ratio (PLR)	210.62±153.47		(31.0–2284.0)
	Glasgow prognostic score (GPS)	0.556±0.748		(0–2)
	Controlling nutritional status (CONUT)	2.49±2.35		(0–12)
	Onodera PNI	45.17±6.95		(14.9–67.9)
Postoperative adjuvant chemotherapy				
	done	137		(15%)
	-Stage II	37		(4%)
	-Stage III	100		(11%)
	not done	749		(85%)

The 5-year RFS rates of patients with stage II and III CRC were 83.8% and 62.7%, respectively. Univariate analysis showed that tumor location, stage, PAC, neutrophil (%), lymphocyte (%), RBC count, Hb, MCV, RDW, platelet count, NLR, and LMR were significantly correlated with RFS (Table 2). Subsequent multivariate analysis showed that tumor location, stage, MCV, NLR, and LMR were significantly correlated with RFS. An MCV ≥ 80.5 fL, NLR ≥ 5.5, and LMR < 3.4 had HRs for disease relapse between 1.39 and 1.93. The proportion of MCV<80.5 fL in patients with right sided CRC (RCRC) and left sided CRC (LCRC) were 24.3% and 14.4%, respectively (p = 0.0002, Table 3). On the other hand, LMR was significantly lower in RCRC than in LCRC (3.08 vs. 3.44, p = 0.0040, Table 3). A subgroups analysis of the tumor location showed that MCV was remained as a prognostic factor: Five year RFS of patients with MCV<80.5 fL and ≧80.5 fL were 87.3% and 73.6%, respectively (p = 0.0147) in RCRC, while those were 85.7% and 68.9%, respectively (p = 0.0027) in LCRC. The prognostic values of CRP, albumin, T-Chol, GPS, CONUT score, and Onodera’s PNI were not significantly correlated with RFS.

**Table 2:** Univariate and multivariate analyses of clinical and pathological parameters for the prediction of relapse-free survival.

				Univariate analysis		Multivariate analysis	
			n	5-yr survival	P	Hazard ratio (95% confidence interval)	P
Age	<65		271	73.2%	0.8905		
	≧65		621	73.4%			
Sex	Male		511	71.4%	0.2319		
	Female		381	75.9%			
Tumor location	Right sided colon		317	77%	**0.0010**	1.11 (0.78–1.58)	0.5414
	Left side colon		299	76.6%		1	
	Rectum		276	65.4%		**1.55 (1.12–2.143)**	**0.0073**
Histological grade	well differentiated		59	72.9%	0.9345		
	moderately differentiated		766	73.5%			
	poorly differentiated		12	73.3%			
	other		55	71.0%			
Stage	II		448	83.8%	**<0.0001**	1	**<0.0001**
	III		444	62.7%		**2.66 (1.93–3.68)**	
Laboratory Data	C-reactive protein (mg/dL)	<0.5	424	74.3%	0.0814		
		≧0.5	249	68.6%			
	Albumin (g/dL)	<2.7	36	62.0%	0.4339		
		≧2.7	856	73.6%			
	Total cholesterol (mg/dL)	<220	758	72.7%	0.2932		
		≧220	134	76.6%			
	White blood cell count (x10^9^/L)	<5.50	325	77.0%	0.1442		
		≧5.50	567	71.2%			
	Neutrophil (%)	<73.7	751	74.6%	**0.0460**		
		≧73.7	141	66.5%			
	Lymphocyte (%)	<14.9	111	65.9%	**0.0412**		
		≧14.9	781	74.4%			
	Monocyte (%)	<8.0	540	75.8%	**0.0446**		
		≧8.0	352	69.3%			
	Red blood cell count (x10^9^/μL)	<3.53	187	66.2%	**0.0093**		
		≧3.53	705	75.0%			
	Hemoglobin (g/dL)	<9.5	154	84.3%	**0.0026**	1	0.1562
		≧9.5	738	71.3%		1.42 (0.87–2.41)	
	Mean corpuscular volume (MCV) (fl)	<80.5	160	86.2%	**<0.0001**	1	**0.0082**
		≧80.5	732	70.4%		**1.93 (1.17–3.32)**	
	Red cell distribution width (RDW)	<18.5	680	71.0%	**0.0058**	1.13 (0.77–1.73)	0.5264
		≧18.5	212	80.9%		1	
	Platelets count (x10^9^/L)	<364	746	71.6%	**0.0105**		
		≧364	146	82.2%			
	Neutrophil-to-lymphocyte ratio (NLR)	<5.5	801	74.5%	**0.0098**	1	**0.0239**
		≧5.5	91	62.7%		**1.62 (1.07–2.40)**	
	Lymphocyte-to- monocyte ratio (LMR)	<3.4	466	70.4%	**0.0234**	**1.39 (1.05–1.86)**	**0.0231**
		≧3.4	426	76.4%		1	
	Platelets-to-lymphocyte ratio (PLR)	<218	607	74.6%	0.1954		
		≧218	285	70.6%			
	Glasgow prognostic score (GPS)	0	404	72.0%	0.9171		
		1–2	269	72.8%			
	Controlling nutritional status (CONUT)	0–4	741	73.4%	0.3973		
		5–12	151	73.1%			
	Onodera PNI	<41.8	252	72.5%	0.3176		
		≧41.8	640	73.7%			
Postoperative adjuvant chemotherapy	done		271	66.1%	**0.0020**	0.95 (0.0–1.28)	0.7293
	not done		621	76.7%		1	

**Table 3:** Relations between MCV, NLR, LMR and tumor location. MCV was significantly smaller in right sided CRC than in left sided CRC, while LMR was significantly lower in the right sided CRC than the left sided CRC.

		Right CRC (n = 317)	Left CRC (n = 575)	P
**MCV**	Median	86.4±8.7	89.2±8.2	**<0.0001**
	<80.5 fL	77 (24.3%)	83 (14.4%)	**0.0002**
	≧80.5 fL	240 (75.7%)	492 (85.6%)	
**NLR**	Median	3.27±2.22	3.23±3.07	0.1170
	<5.5	283 (89.3%)	518 (90.1%)	0.7012
	≧5.5	34 (10.7%)	57 (9.9%)	
**LMR**	Median	3.51±3.70	3.62±1.68	**0.0040**
	<3.4	189 (59.6%)	277 (48.2%)	**0.0011**
	≧3.4	128 (40.4%)	298 (51.8%)	

The 5-year OS of patients with stage II and III CRC were 85.1% and 72.0%, respectively. Univariate analysis showed that age, sex, stage, CRP, lymphocyte (%), monocyte (%), RBC count, Hb, MCV, RDW, platelet count, NLR, LMR, CONUT, and Onodera PNI were significantly correlated with OS (Table 4). The multivariate analysis showed that sex, stage, RBC count, and RDW were significantly correlated with OS.

**Table 4:** Univariate and multivariate analyses of clinical and pathological parameters for the prediction of overall survival.

				Univariate analysis		Multivariate analysis	
			n	5-yr survival	P	Hazard ratio (95% confidence interval)	P
Age	<65		271	82.9%	**0.0213**	1	0.0891
	≧65		621	76.7%		1.35 (0.95–1.97)	
Sex	Male		511	75.8%	**0.0019**	**1.58 (1.14–2.20)**	**0.0058**
	Female		381	82.3%		1	
Tumor location	Right sided colon		317	79%	0.2737		
	Left side colon		299	81.2%			
	Rectum		276	74.9%			
Histological grade	well differentiated		59	77.7%	0.5451		
	moderately differentiated		766	79.1%			
	poorly differentiated		12	60.6%			
	other		55	76.9%			
Stage	II		448	85.1%	**<0.0001**	1	**<0.0001**
	III		444	72.0%		**2.00 (1.46–2.77)**	
Laboratory Data	C-reactive protein (mg/dL)	<0.5	424	79.9%	**0.0353**	1	0.3984
		≧0.5	249	73.0%		1.15 (0.83–1.60)	
	Albumin (g/dL)	<2.7	36	70.7%	0.1001		
		≧2.7	856	79.0%			
	Total cholesterol (mg/dL)	<220	758	77.6%	0.1096		
		≧220	134	84.0%			
	White blood cell count (x10^9^/L)	<5.50	325	81.3%	0.1030		
		≧5.50	567	77.0%			
	Neutrophil (%)	<73.7	751	79.2%	0.0728		
		≧73.7	141	75.6%			
	Lymphocyte (%)	<14.9	111	71.4%	**0.0094**		
		≧14.9	781	79.6%			
	Monocyte (%)	<8.0	540	82.6%	**0.0011**		
		≧8.0	352	72.4%			
	Red blood cell count (x10^9^/μL)	<3.53	187	68.7%	**<0.0001**	**1.80 (1.24–2.59)**	**0.0024**
		≧3.53	705	81.4%		1	
	Hemoglobin (g/dL)	<9.5	154	77.4%	0.8685		
		≧9.5	738	78.9%			
	Mean corpuscular volume (MCV) (fl)	<80.5	160	86.6%	**0.0019**	1.40 (0.85–2.41)	0.1830
		≧80.5	732	76.9%		1	
	Red cell distribution width (RDW)	<18.5	680	77.9%	**0.0408**	1	**0.0477**
		≧18.5	212	81.0%		**1.56 (1.00–2.49)**	
	Platelets count (x10^9^/L)	<364	746	77.3%	**0.0386**		
		≧364	146	85.5%			
	Neutrophil-to-lymphocyte ratio (NLR)	<5.5	801	79.8%	**0.0069**	1.35(0.84–2.11)	0.2062
		≧5.5	91	68.1%		1	
	Lymphocyte-to- monocyte ratio (LMR)	<3.4	466	74.4%	**0.0010**	1.30 (0.91–1.86)	0.1495
		≧3.4	426	83.0%		1	
	Platelets-to-lymphocyte ratio (PLR)	<218	607	79.2%	0.5393		
		≧218	285	77.2%			
	Glasgow prognostic score (GPS)	0	404	79.6%	0.1788		
		1–2	269	73.8%			
	Controlling nutritional status (CONUT)	0–4	741	80.7%	**0.0003**	1	0.6614
		5–12	151	67.4%		1.13 (0.66–1.94)	
	Onodera PNI	<41.8	252	69.2%	**0.0002**	1.10 (0.67–1.74)	0.7026
		≧41.8	640	82.0%		1	
Postoperative adjuvant chemotherapy	done		271	78.6%	0.6399		
	not done		621	78.7%			

**Fig 1:**
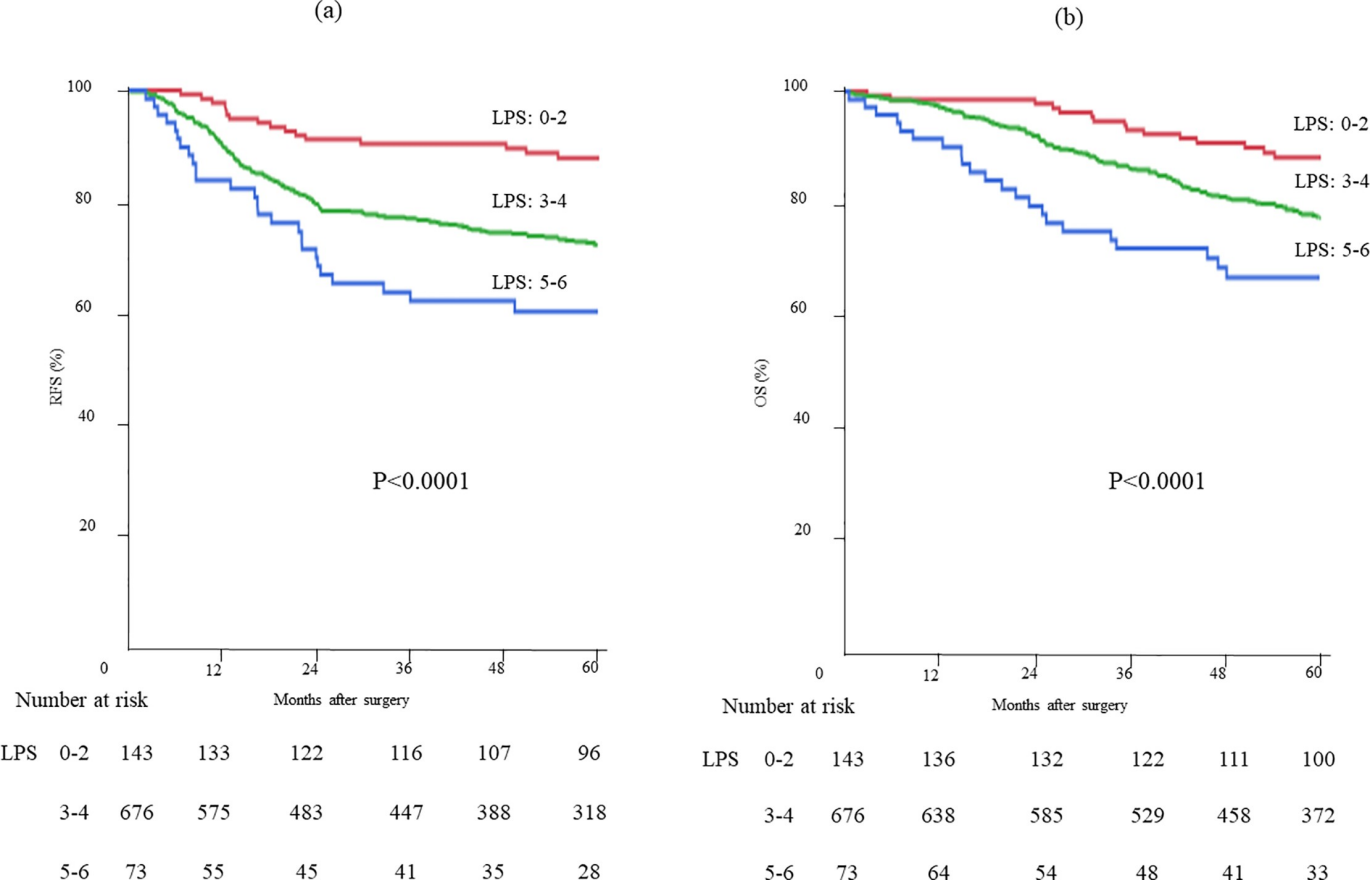
Outcomes of all patients based on laboratory prognostic score (LPS): The RFS (a) and OS (b) curves. **The RFS(a) and OS (b) were well stratified by LPS**.

**Fig 2:**
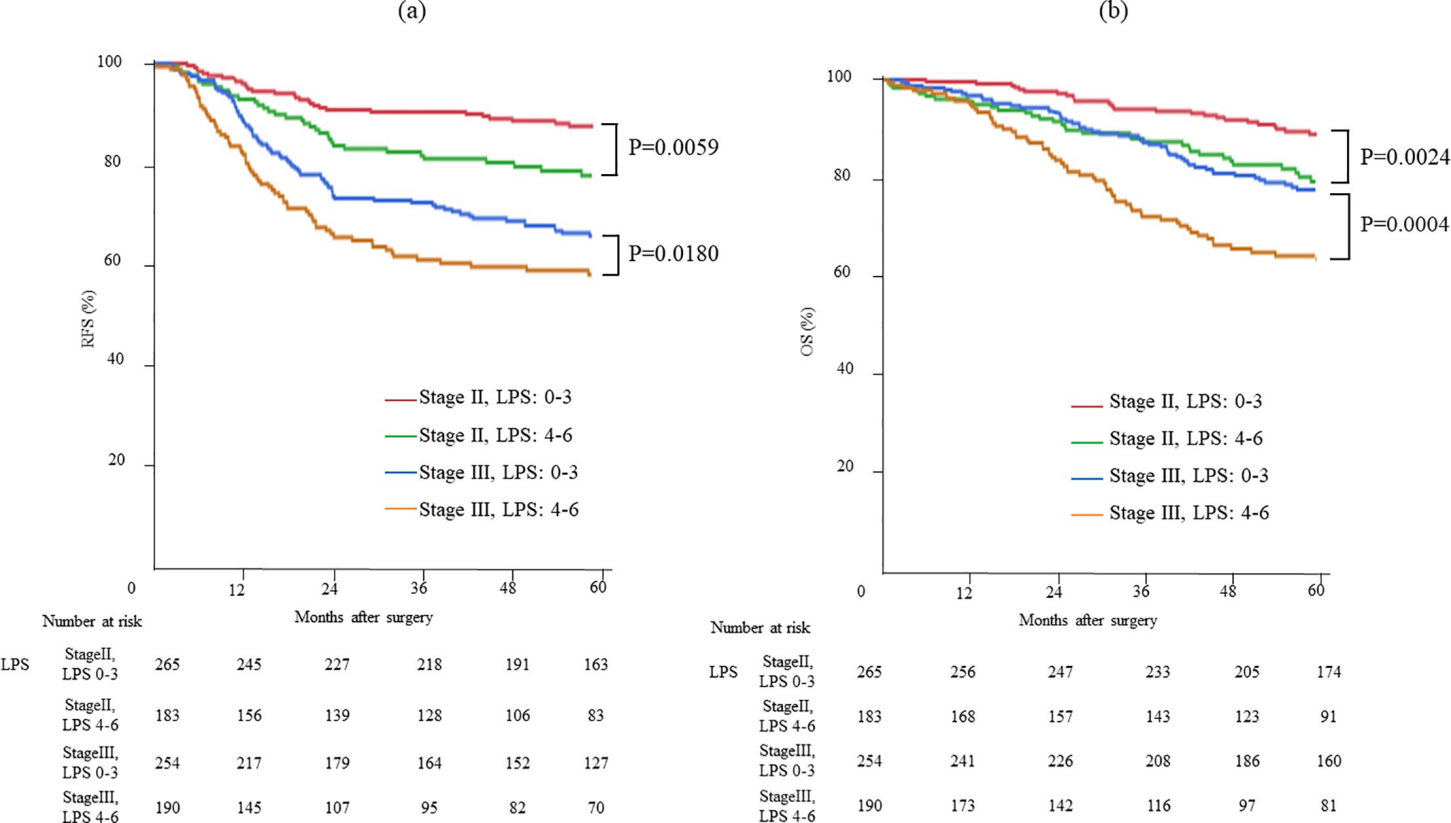
Outcomes of all patients according to stage and laboratory prognostic score (LPS): RFS (a) and OS (b) curves. **The RFS (a) and OS (b) classified by stage and LPS were well stratified by LPS in stage II and III**.

**Fig 3:**
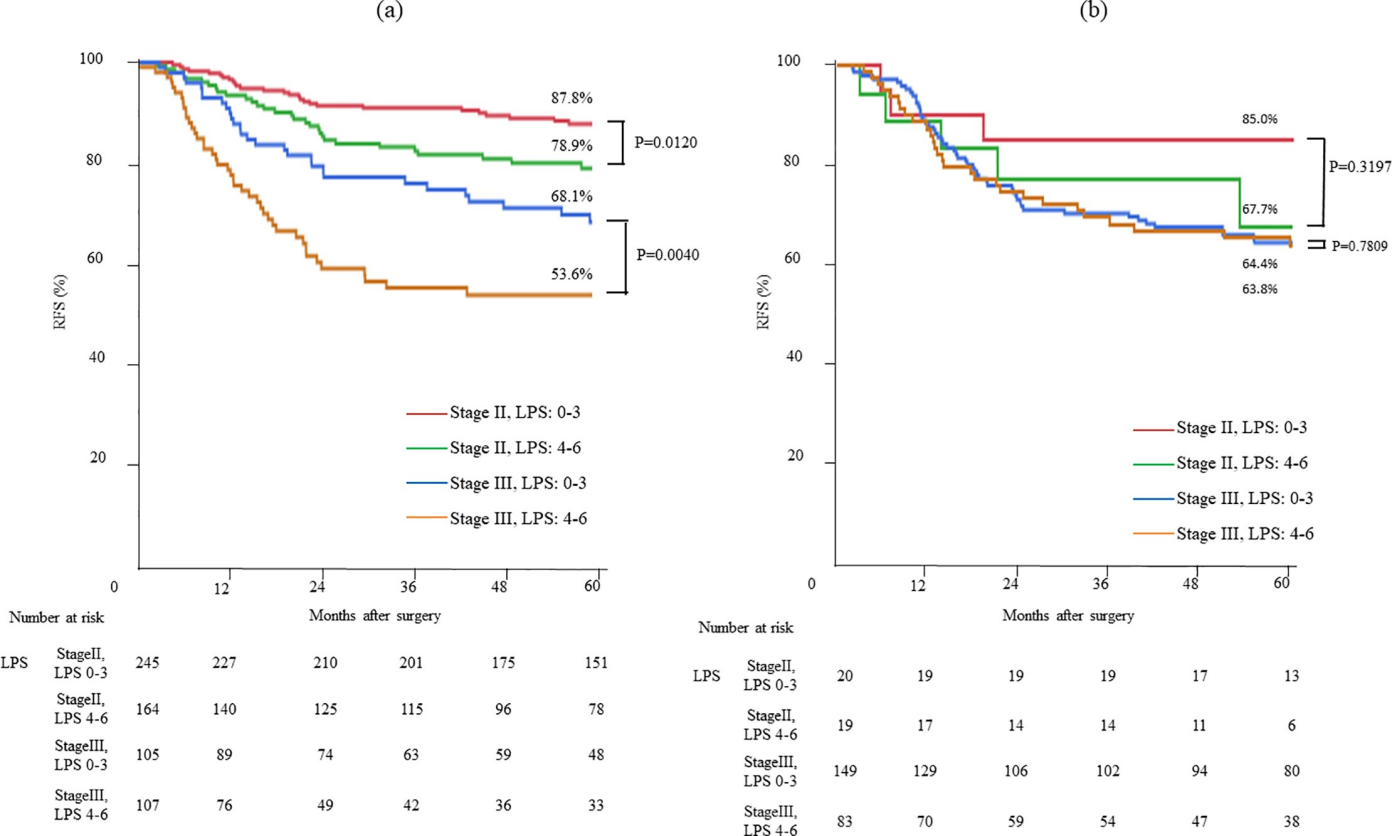
RFS curves according to stage and LPS: (a) patients without postoperative adjuvant chemotherapy (PAC), (b) patients with PAC. **The RFS of patients who did not receive PAC was significantly stratified by LPS of 0–3 and 4–6 (Fig 3A); however, the prognostication by LPS was less remarkable in patients who received PAC (Fig 3B)**.

The RFS and OS of the study patients were well stratified by an LPS between 0 and 6 (Fig 1). The RFS and OS of stage II and III CRC patients were significantly inferior with an LPS of 4–6 than with an LPS of 0–3 (Fig 2). Additional subgroup analysis based on PAC was performed (Fig 3A and 3B). Although the RFS of the patients who did not receive PAC was significantly stratified by the LPS, prognostication by LPS was less remarkable in patients who received PAC. The RFS of stage II CRC patients who received PAC was inferior to that of patients who did not receive PAC, regardless of the LPS. On the other hand, for the stage III CRC patients with an LPS of 4–6, the 5-year RFS tended to be better in those who received PAC than in those who did not receive PAC (63.8% vs. 53.6%, p = 0.0665).

## Discussion

Our findings demonstrated that preoperative MCV ≥ 80.5 fL, NLR ≥ 5.5, and LMR < 3.4 were significant predictors of poor RFS in stage II and III CRC patients who underwent curative resection. The RFS and OS of stage II and III patients were significantly stratified by the LPS, which was developed from the three indices.

The MCV indicates the volume of RBCs and is frequently used for the diagnosis of megaloblastic or iron-deficiency anemia. Meanwhile, recent studies found a prognostic implication of MCV in esophageal and liver cancer [19–21]. Our group previously reported that MCV was a prognostic factor for RFS in patients who underwent R0 resection for stage I/II/III CRC, independent of the tumor stage [22]; a low MCV or microcytosis (MCV < 80 fL) was associated with favorable outcomes. Schneider C et al. reported that Hb and MCV dropped shortly before the CRC diagnosis, and low MCV was weakly associated with survival of patients with CRC [23]. CRC is often accompanied by iron-deficiency anemia, which leads to decreased MCV. The mechanism responsible for the association between MCV and disease relapse is unknown, although several hypotheses have been proposed. First, oxidative stress had been implicated in a variety of chronic diseases, and the antioxidant capacity of the body had been related with the size of the circulating RBCs [24]. Because an elevated MCV or macrocytosis may reflect structural or functional disorders of RBCs, its unfavorable effects on relapse after R0 resection for CRC can be explained by a disturbed antioxidant capacity. Additionally, impaired deformability of the RBCs from high oxidative stress can damage the microcirculation and oxygen delivery to tissues [25, 26]. Second, macrocytosis may be a sign of disturbed hematopoiesis due to bone marrow dysfunction. Bone marrow-derived mesenchymal stem cells have been reported to play a critical role in the repair of several damaged vital organs [27]. Mahmoud et al. reported that unexplained macrocytosis in elderly patients was related with dysplastic or pathologic findings consistent with myelodysplastic syndrome [28]. Ueda et al. investigated the blood cell components in patients with acute decompensated heart failure and showed that the WBC and platelet counts were significantly lower in the macrocytic group than in the non-macrocytic group [29]. Third, although our study showed that albumin, T-Chol, GPS, CONUT score, and Onodera’s PNI were not significantly associated with RFS, the patients’ nutrition can be related with MCV. A relatively high MCV may be a surrogate marker of folic acid or vitamin B12 deficiency, which the present study did not investigate. Fourth, crystal osmotic pressure, which is closely related with MCV and is mainly determined by the serum concentrations of glucose, amino acids, and electrolytes, regulates the RBC size [30]. These factors that can affect RBC volume were not investigated in the present study. Fifth, tumor location can be related with MCV. Väyrynen et al. reported that proximal tumor location was associated with predominant microcytic anemia [31]. Several studies showed that the prognosis of patients who underwent curative resection for RCRC was favorable, compared with that for LCRC [32–34]. Our study showed that the proportion of MCV<80.5 fL in patients with RCRC was significantly higher than those with LCRC, however, MCV was remained as a prognostic factor in a subgroup analysis of the tumor location. Sixth, endothelial function can be related with MCV. Endothelial dysfunction had been considered as one of the important mechanisms of the association between cardiovascular disease and chronic kidney disease [35]. Flow-mediated dilation, which is a noninvasive examination to assess vascular function, was demonstrated by Solak et al. to have an inverse association with MCV, independent of insulin resistance and inflammation [36].

The present study showed that the NLR was the second important prognostic factor among the blood cell markers. Tumor-associated inflammatory cytokines and mediators may mediate inflammatory responses, which lead to tumor growth, infiltration, and metastasis. Lymphopenia is a surrogate marker for impaired cell-mediated immunity, whereas neutrophilia is acknowledged as a response to systematic inflammation [37]. Lymphocytes participate in cytotoxic cell death and inhibition of tumor cell proliferation and migration [38, 39]. The NLR, which is equivalent to the number of neutrophils divided by the number of lymphocytes, had been widely investigated as an indicator of systemic inflammatory response in CRC. Many studies showed that a high NLR, with cutoff values ranging between 2 and 5, was associated with poor long-term outcomes in patients with CRC [2, 5, 9, 40–45].

Monocytes can promote tumor progression and metastasis [46, 47]. Tumor-associated macrophages (TAM) that are derived from circulating monocytes can suppress adaptive immunity and promote angiogenesis, invasion, and migration [48]. Increased circulating monocytes may reflect increased levels of TAM and worse prognosis. However, the circulating monocyte level had not been widely investigated as a biomarker of CRC. The LMR is the ratio of the absolute lymphocyte count to the absolute monocyte count in blood. Stotz et al. showed the prognostic value of preoperative LMR (2.83) in patients with stage III CRC [49], and was in line with the results of studies by Song Y et al [2] and Chan JC et al [12]. Recent studies have indicated that low LMR, with cutoff values ranging between 3.0 and 4.8, was associated with poor long-term outcomes in patients with CRC [2, 12, 49–53].

The present study revealed that MCV, NLR, and LMR were superior to the conventional blood markers, including CRP, albumin, T-Chol, GPS, CONUT score, and Onodera’s PNI [7, 14, 15], in predicting RFS. This result suggested that these conventional prognostic markers were less useful for the prognostication of patients who underwent R0 resection for stage II and III CRC. Chan JC et al. reported similar results of ours about GPS [12]. The discrepancy among the studies may be derived from the differences in patient selection, tumor stage, treatment, and outcomes measurement.

We developed the LPS for easy application of these three indices (i.e., MCV ≥ 80.5 fL, NLR ≥ 5.5, and LMR < 3.4) for prognostication. The RFS of stage II and III CRC patients was significantly inferior with an LPS of 4–6 than with an LPS of 0–3 (Fig 2). Additional subgroup analysis according to PAC showed that the RFS of patients who did not receive PAC was significantly stratified by LPS of 0–3 and 4–6 (Fig 3A); however, the prognostication by LPS was less remarkable in patients who received PAC (Fig 3B). The RFS of stage II CRC patients who received PAC was inferior to those who did not receive PAC, regardless of the LPS. This can be partly explained by the fact that the stage II CRC patients who received PAC had several poor prognostic factors. On the other hand, the 5-year RFS of stage III CRC patients with an LPS of 4–6 tended to be better in those who received PAC than in those who did not receive PAC (p = 0.065). The result suggested the protective effect of PAC in patients with LPS 4–6 and the usefulness of LPS in predicting the response to PAC in stage III CRC.

The current study had several limitations. First, it was a retrospective single-institutional study, although it included a large and homogenous dataset. The unknown backgrounds that may affect MCV, lymphocytes, and monocytes may have led to a selection bias. Second, patients who received PAC were included and comprised 30% of the entire cohort. PAC modifies the RFS and can act as an important confounder. Patient choice, age, comorbidities, and postoperative complications can affect the performance of PAC. Nevertheless, our additional subgroup analysis based on PAC showed that the RFS of the patients who did not receive PAC was significantly different between LPS 0–3 and 4–6. Third, the patients’ performance status (e.g., the American Society of Anesthesiologists score), surgical approach (open or laparoscopic), and postoperative complications, all of which can affect the RFS, were not investigated in this study. Fourth, CRP was measured in only 75% of the entire cohort, because our surgical department did not have routine preoperative measurement of CRP. Comparison of the prognostic ability among MCV, NLR, LMR, and GPS will be necessary in future studies with full measurement. Despite these limitations, the results of our study may be clinically relevant in the context of pre- and postoperative treatment or surveillance planning, because MCV, NLR, and LMR can be obtained from routine blood cell examination, which is readily available and inexpensive.

## Conclusions

MCV, NLR, and LMR were useful blood cell markers that significantly predicted the RFS of patients who underwent R0 resection for stage II and III CRC, independent of tumor stage. The LPS developed from these three factors can predict response to PAC in stage III CRC. Future prospective studies are required to validate these results.
